# Silicon Nanoparticles Modulate C:N:P Homeostasis and the Efficiencies of Nutrient Uptake, Translocation, and Use in Sugarcane Under Calcium Deficiency and Sufficiency

**DOI:** 10.3390/plants15060971

**Published:** 2026-03-21

**Authors:** João Victor da Silva Santos, Milton Garcia Costa, João Vitor Silva e Silva, Francisco Sales Ferreira dos Santos, Renato de Mello Prado

**Affiliations:** Laboratory of Plant Nutrition, School of Agricultural and Veterinary Science, São Paulo State University, Via de Acesso Professor Paulo Donato Castelane Castellane S/N-Vila Industrial, Jaboticabal 14884-900, São Paulo, Brazil; milton.costa@unesp.br (M.G.C.); vitor.e@unesp.br (J.V.S.e.S.); sales.ferreira@unesp.br (F.S.F.d.S.J.); rm.prado@unesp.br (R.d.M.P.)

**Keywords:** nutritional stress, nutrient use efficiency, silicon nanoparticles, calcium deficiency, stoichiometric homeostasis

## Abstract

Calcium (Ca) deficiency is a major nutritional constraint for sugarcane, impairing stoichiometric homeostasis and biomass accumulation. In this context, silicon dioxide nanoparticles (nSiO_2_) have emerged as a promising alternative due to their high reactivity and potential to modulate mineral homeostasis. This study evaluated the effects of nSiO_2_ on C:N:P:Si homeostasis and on nutrient uptake, translocation, and use efficiencies in sugarcane plants grown under Ca deficiency and sufficiency. The experiment was conducted in a greenhouse using a 2 × 2 factorial design, with two Ca conditions (0 and 3 mmol L^−1^) and two nSiO_2_ conditions (0 and 1.77 mmol L^−1^ of Si), with four replications. Calcium deficiency reduced nutrient accumulation and nutritional efficiencies of several macro- and micronutrients, disrupted stoichiometric ratios, and decreased shoot dry mass. The application of nSiO_2_ under Ca deficiency increased Si concentration and accumulation along with other nutrients, reduced C:Si ratios, enhanced nutrient uptake, translocation, and use efficiencies, and resulted in increased shoot biomass. Under Ca-sufficient conditions, nSiO_2_ promoted nutritional adjustments and improved nutrient efficiencies but did not affect biomass production. Overall, the results demonstrate that nSiO_2_ acts as a nutritional modulator and is more effective in mitigating the adverse effects of Ca deficiency through stoichiometric rebalancing and improved nutrient use efficiencies.

## 1. Introduction

Sugarcane (*Saccharum officinarum* L.) is one of the most strategic agricultural crops worldwide, supporting high-value chains linked to sugar, ethanol, and bioelectricity production, as well as coproducts of increasing relevance to the bioeconomy [1]. In tropical systems, where sugarcane occupies extensive areas and is managed intensively, gains in productivity and yield stability are directly dependent on nutritional management and resource-use efficiency, particularly under scenarios of increasing climate variability and edaphic constraints [2,3].

Among macronutrients, calcium (Ca) plays a central role in the structural and functional organization of plant tissues [4]. Reduced Ca availability in the growth medium is rapidly reflected in lower Ca concentrations in shoots and roots, as this element has limited phloem mobility and relies strongly on transpirational flow and xylem transport to reach growing organs [5,6]. This decline in Ca status triggers disorders related to membrane and cell wall integrity—particularly through interactions with pectins—and compromises Ca^2+^-dependent signaling processes, affecting root growth, transporter activity, and metabolic coordination under nutritional stress [7,8].

Consequently, Ca deficiency is not limited to Ca itself but can induce reprogramming of mineral homeostasis and elemental balance, affecting C, N, and P stoichiometry and the allocation of resources between maintenance and growth [9]. Alterations in C:N:P ratios are especially relevant, as they integrate N and P supply and assimilation, metabolic costs of biomass synthesis, and nutrient-to-biomass conversion efficiency. This framework has been widely used to explain performance losses in sugarcane under stress and nutritional imbalances [10,11]. In parallel, disturbances in nutritional homeostasis are often expressed as changes in nutrient accumulation and in uptake, translocation, and use efficiencies, reflecting compensatory adjustments in the root system and nutrient partitioning to sustain vital functions [12].

In this context, nanotechnology has emerged as a promising alternative to enhance the efficiency and precision of input supply, and silicon dioxide nanoparticles (nSiO_2_) have gained attention due to their high surface area, reactivity, and potential to modulate physiological and nutritional responses [13,14]. Recent evidence indicates that Si nanoparticles can influence nutrient acquisition and transport, interact with signaling pathways—including Ca^2+^ signaling—and adjust defense mechanisms and photochemical performance, thereby directly affecting nutritional [15,16]. Moreover, silicon has been shown to mitigate symptoms and limitations associated with Ca deficiency and, more broadly, to reduce damage caused by mineral deficiencies, suggesting a role in reorganizing nutritional balance and supporting elemental homeostasis [17,18].

Despite these advances, the mechanistic understanding of how nSiO_2_ contributes to restoring C:N:P balance and to the integrated reconfiguration of nutrient concentrations, accumulations, and uptake, translocation, and use efficiencies in sugarcane under Ca restriction remains limited. Thus, it is pertinent to evaluate the hypotheses that (i) Ca deficiency disrupts nutritional status and C:N:P homeostasis, leading to biomass loss; (ii) nSiO_2_ acts as a mitigating agent by promoting the reestablishment of nutritional balance and sustaining growth, particularly under Ca deficiency; and (iii) the effects of nSiO_2_ application are not restricted to stress conditions but may also extend benefits under adequate Ca supply.

Accordingly, the objective of this study was to evaluate whether nSiO_2_ modulates C:N:P:Si homeostasis, as well as nutrient uptake, translocation, and use efficiencies and dry mass accumulation in sugarcane plants grown under Ca deficiency and sufficiency. The results provide evidence that Ca deficiency broadly disrupts sugarcane nutritional balance, affecting C:N:P stoichiometry, nutrient fluxes, and nutritional efficiencies, with direct impacts on biomass accumulation. Furthermore, they demonstrate that nSiO_2_ represents a promising tool to restore nutritional balance, especially under Ca deficiency, reinforcing its potential to improve management strategies and enhance fertilizer use efficiency in sugarcane production systems.

## 2. Results

### 2.1. Nutrient Concentrations

**Figure 1:**
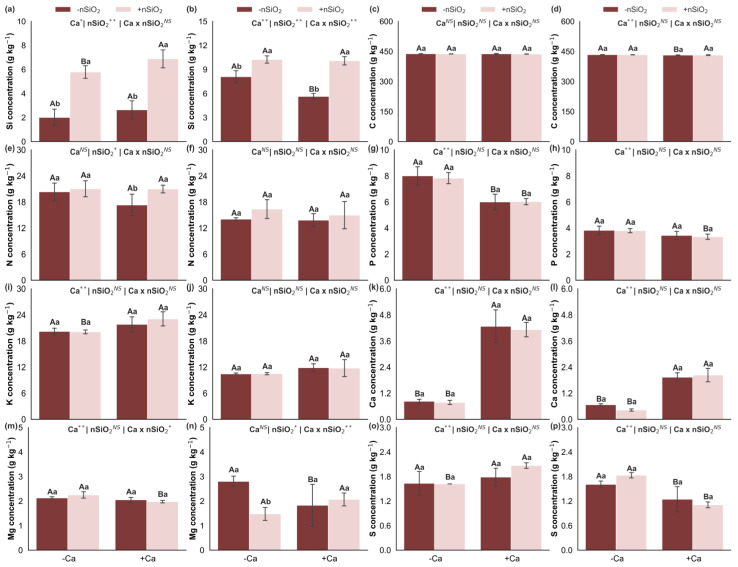
Silicon (**a**,**b**), carbon (**c**,**d**), nitrogen (**e**,**f**), phosphorus (**g**,**h**), potassium (**i**,**j**), calcium (**k**,**l**), magnesium (**m**,**n**), and sulfur (**o**,**p**) concentrations in the shoot and roots of sugarcane plants grown under calcium deficiency (−Ca) or sufficiency (+Ca), combined with the absence (−nSiO_2_) or presence (+nSiO_2_) of silicon nanoparticle application. Bars represent means (*n* = 4) and error bars indicate the standard deviation (SD). Identical uppercase letters indicate no significant differences between Ca supply conditions, whereas identical lowercase letters indicate no significant differences between nSiO_2_ application conditions. *, ** and NS represent significance at 0.05, 0.01, and non-significant, respectively.

Under Ca deficiency, in the absence of nSiO_2_ (−nSiO_2_), Ca deficiency led to reduced Ca concentrations in both shoots and roots, accompanied by increased P concentration in the shoots and increased Si, C, Mg, and S concentrations in the roots. In contrast, the concentrations of Si, C, N, K, Mg, and S in the shoots, as well as N, P, K, and Mg in the roots, did not show significant variation (Figure 1). Under Ca-deficient conditions, the application of nSiO_2_ (+nSiO_2_) increased Si concentrations in both shoots and roots and decreased Mg concentration in the roots, with no significant effects on C, N, P, K, Ca, Mg, and S in the shoots or on C, N, P, K, Ca, and S in the roots (Figure 1). Under Ca-sufficient conditions, nSiO_2_ application increased Si and N concentrations in the shoots and Si concentration in the roots, whereas the remaining concentrations (C, P, K, Ca, Mg, and S in the shoots; C, N, P, K, Ca, Mg, and S in the roots) did not respond significantly (Figure 1).

**Figure 2:**
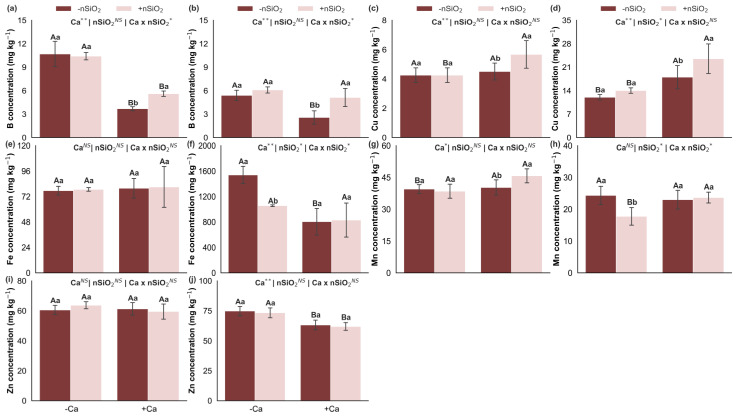
Boron (**a**,**b**), copper (**c**,**d**), iron (**e**,**f**), manganese (**g**,**h**) and zinc (**i**,**j**) concentrations in the shoot and roots of sugarcane plants grown under calcium deficiency (−Ca) or sufficiency (+Ca), combined with the absence (−nSiO_2_) or presence (+nSiO_2_) of silicon nanoparticle application. Bars represent means (*n* = 4) and error bars indicate the standard deviation (SD). Identical uppercase letters indicate no significant differences between Ca supply conditions, whereas identical lowercase letters indicate no significant differences between nSiO_2_ application conditions. *, ** and NS represent significance at 0.05, 0.01, and non-significant, respectively.

Under Ca deficiency, in the absence of nSiO_2_, Ca deficiency increased B concentration in the shoots and B, Fe, and Zn concentrations in the roots, while decreasing Mn concentration in the shoots and Cu concentration in the roots (Figure 2). The concentrations of Cu, Fe, and Zn in the shoots and Mn in the roots were not affected (Figure 2). In Ca-deficient plants, the application of nSiO_2_ was associated with reduced Fe and Mn concentrations in the roots, with no changes in B, Cu, Fe, Mn, and Zn in the shoots or in B, Cu, and Zn in the roots (Figure 2). Under Ca-sufficient conditions, nSiO_2_ application increased B, Cu, and Mn concentrations in the shoots and B and Cu concentrations in the roots, whereas Fe and Zn in the shoots and Fe, Mn, and Zn in the roots did not differ significantly (Figure 2).

### 2.2. C, N, P and Si Stoichiometry

**Figure 3:**
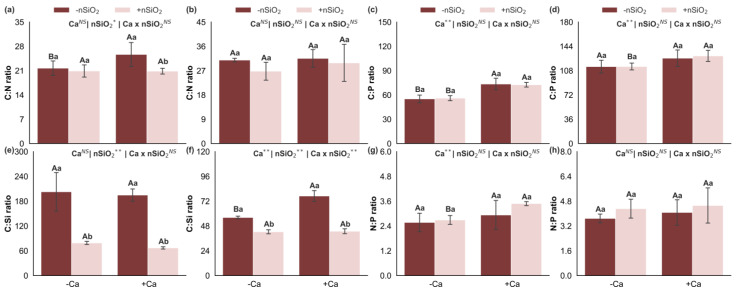
C:N (**a**,**b**), C:P (**c**,**d**), C:Si (**e**,**f**) and N:P (**g**,**h**) ratios in the shoot and roots of sugarcane plants grown under calcium deficiency (−Ca) or sufficiency (+Ca), combined with the absence (−nSiO_2_) or presence (+nSiO_2_) of silicon nanoparticle application. Bars represent means (*n* = 4) and error bars indicate the standard deviation (SD). Identical uppercase letters indicate no significant differences between Ca supply conditions, whereas identical lowercase letters indicate no significant differences between nSiO_2_ application conditions. *, ** and NS represent significance at 0.05, 0.01, and non-significant, respectively.

Under Ca deficiency, in the absence of nSiO_2_, Ca deficiency decreased the C:N and C:P ratios in the shoots and the C:P and C:Si ratios in the roots, with no significant effects on C:Si and N:P ratios in the shoots or on C:N and N:P ratios in the roots (Figure 3). Under Ca-deficient conditions, the application of nSiO_2_ reduced the C:Si ratio in both shoots and roots, whereas C:N, C:P, and N:P ratios remained unchanged in both compartments (Figure 3). In Ca-sufficient plants, nSiO_2_ application also decreased the C:N and C:Si ratios in the shoots and the C:Si ratio in the roots; in contrast, C:P and N:P ratios in the shoots and C:N, C:P, and N:P ratios in the roots did not show significant responses (Figure 3).

### 2.3. Nutrient Accumulations

**Figure 4:**
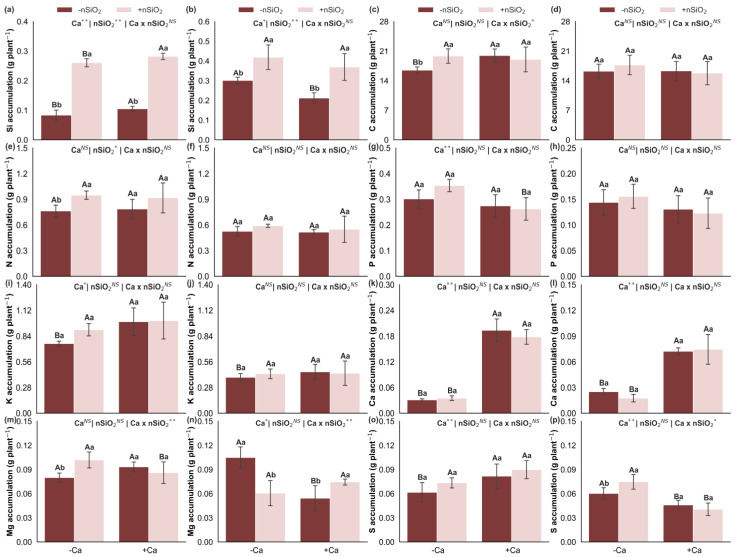
Silicon (**a**,**b**), carbon (**c**,**d**), nitrogen (**e**,**f**), phosphorus (**g**,**h**), potassium (**i**,**j**), calcium (**k**,**l**), magnesium (**m**,**n**), and sulfur (**o**,**p**) accumulations in the shoot and roots of sugarcane plants grown under calcium deficiency (−Ca) or sufficiency (+Ca), combined with the absence (−nSiO_2_) or presence (+nSiO_2_) of silicon nanoparticle application. Bars represent means (*n* = 4) and error bars indicate the standard deviation (SD). Identical uppercase letters indicate no significant differences between Ca supply conditions, whereas identical lowercase letters indicate no significant differences between nSiO_2_ application conditions. *, ** and NS represent significance at 0.05, 0.01, and non-significant, respectively.

Under Ca deficiency, in the absence of nSiO_2_, Ca deficiency was associated with reduced accumulation of Si, C, K, Ca, and S in the shoots and of K and Ca in the roots (Figure 4). In contrast, Si, Mg, and S accumulation increased in the roots, whereas the accumulation of N, P, and Mg in the shoots and of C, N, and P in the roots did not differ significantly (Figure 4). Under Ca-deficient conditions, the application of nSiO_2_ increased the accumulation of Si, C, N, and Mg in the shoots and of Si and S in the roots, while decreasing Mg accumulation in the roots. The accumulation of P, K, Ca, and S in the shoots and of C, N, P, K, and Ca in the roots showed no significant differences (Figure 4). Under Ca-sufficient conditions, nSiO_2_ application increased Si accumulation in the shoots and Si and Mg accumulation in the roots, with no significant responses observed for the remaining nutrients evaluated in the shoots (C, N, P, K, Ca, Mg, and S) and in the roots (C, N, P, K, Ca, and S) (Figure 4).

**Figure 5:**
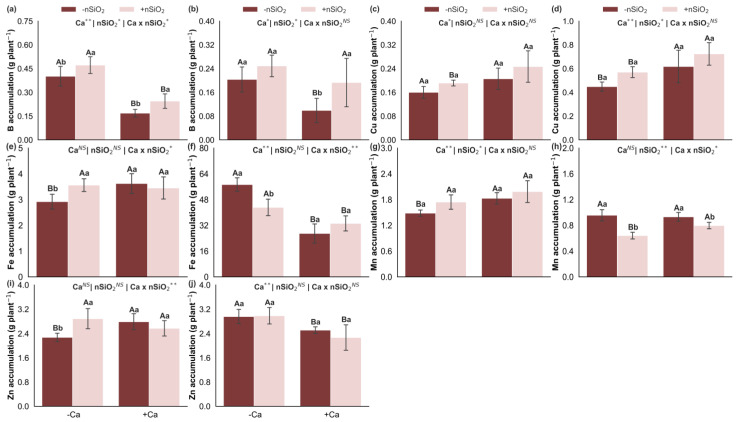
Boron (**a**,**b**), copper (**c**,**d**), iron (**e**,**f**), manganese (**g**,**h**) and zinc (**i**,**j**) accumulations in the shoot and roots of sugarcane plants grown under calcium deficiency (−Ca) or sufficiency (+Ca), combined with the absence (−nSiO_2_) or presence (+nSiO_2_) of silicon nanoparticle application. Bars represent means (*n* = 4) and error bars indicate the standard deviation (SD). Identical uppercase letters indicate no significant differences between Ca supply conditions, whereas identical lowercase letters indicate no significant differences between nSiO_2_ application conditions. *, ** and NS represent significance at 0.05, 0.01, and non-significant, respectively.

Under Ca deficiency, in the absence of nSiO_2_, Ca deficiency increased B accumulation in the shoots and B, Fe, and Zn accumulation in the roots, while resulting in decreased accumulation of Fe, Mn, and Zn in the shoots and of Cu in the roots. The accumulation of Cu in the shoots and Mn in the roots did not differ significantly (Figure 5). In Ca-deficient plants, the application of nSiO_2_ increased Fe and Zn accumulation in the shoots and reduced Fe and Mn accumulation in the roots, with no effects on the accumulation of B, Cu, and Mn in the shoots or of B, Cu, and Zn in the roots (Figure 5). Under Ca-sufficient conditions, nSiO_2_ application increased B accumulation in both shoots and roots and decreased Mn accumulation in the roots, whereas the accumulation of Cu, Fe, Mn, and Zn in the shoots and of Cu, Fe, and Zn in the roots did not differ significantly (Figure 5).

### 2.4. Nutrient Uptake Efficiency

**Figure 6:**
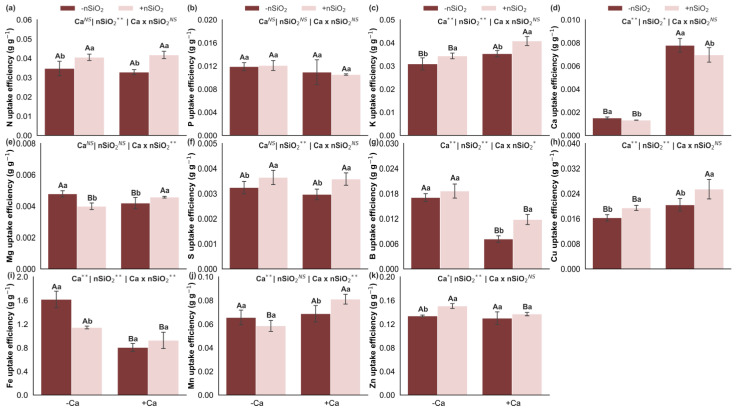
Nitrogen (**a**), phosphorus (**b**), potassium (**c**), calcium (**d**), magnesium (**e**), sulfur (**f**), boron (**g**), copper (**h**), iron (**i**), manganese (**j**) and zinc (**k**) uptake efficiency in sugarcane plants grown under calcium deficiency (−Ca) or sufficiency (+Ca), combined with the absence (−nSiO_2_) or presence (+nSiO_2_) of silicon nanoparticle application. Bars represent means (*n* = 4) and error bars indicate the standard deviation (SD). Identical uppercase letters indicate no significant differences between Ca supply conditions, whereas identical lowercase letters indicate no significant differences between nSiO_2_ application conditions. *, ** and NS represent significance at 0.05, 0.01, and non-significant, respectively.

Under Ca deficiency, in the absence of nSiO_2_, there was a decrease in the uptake efficiencies of K, Ca, and Cu, an increase in the uptake efficiencies of Mg, B, and Fe, and no changes in the uptake efficiencies of N, P, S, Mn, and Zn (Figure 6). Additionally, under Ca-deficient conditions, the application of nSiO_2_ decreased the uptake efficiencies of Ca, Mg, and Fe, increased the uptake efficiencies of N, K, S, Cu, and Zn, and did not alter the uptake efficiencies of P, B, and Mn (Figure 6). Under Ca-sufficient conditions, nSiO_2_ application resulted in a decrease in Ca uptake efficiency, an increase in the uptake efficiencies of N, K, Mg, S, B, Cu, and Mn, and no response in the uptake efficiencies of P, Fe, and Zn (Figure 6).

### 2.5. Nutrient Translocation Efficiency

**Figure 7:**
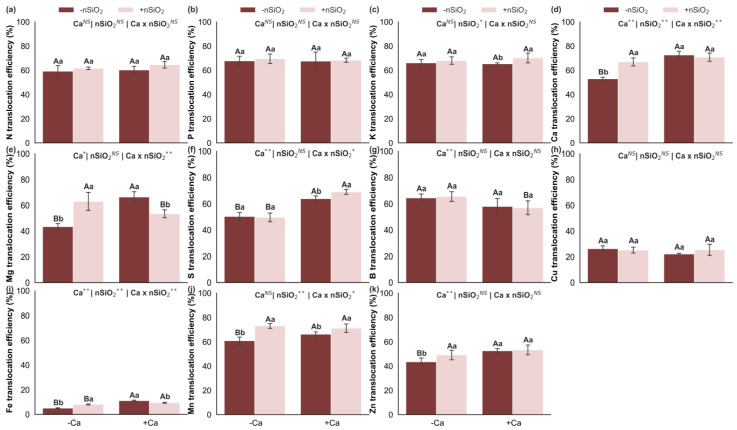
Nitrogen (**a**), phosphorus (**b**), potassium (**c**), calcium (**d**), magnesium (**e**), sulfur (**f**), boron (**g**), copper (**h**), iron (**i**), manganese (**j**) and zinc (**k**) translocation efficiency in sugarcane plants grown under calcium deficiency (−Ca) or sufficiency (+Ca), combined with the absence (−nSiO_2_) or presence (+nSiO_2_) of silicon nanoparticle application. Bars represent means (*n* = 4) and error bars indicate the standard deviation (SD). Identical uppercase letters indicate no significant differences between Ca supply conditions, whereas identical lowercase letters indicate no significant differences between nSiO_2_ application conditions. *, ** and NS represent significance at 0.05, 0.01, and non-significant, respectively.

Under Ca deficiency, in the absence of nSiO_2_, reduced translocation efficiencies were observed for Ca, Mg, S, Fe, Mn, and Zn, whereas the translocation efficiencies of N, P, K, B, and Cu were not affected (Figure 7). In Ca-deficient plants, the application of nSiO_2_, compared with its absence, increased the translocation efficiencies of Ca, Mg, Fe, Mn, and Zn, with no effects on the translocation efficiencies of N, P, K, S, B, and Cu (Figure 7). Under Ca-sufficient conditions, nSiO_2_ application resulted in increased translocation efficiencies of K, S, and Mn, decreased translocation efficiencies of Mg and Fe, and no response for the translocation efficiencies of N, P, Ca, B, Cu, and Zn (Figure 7).

### 2.6. Nutrient Use Efficiency

**Figure 8:**
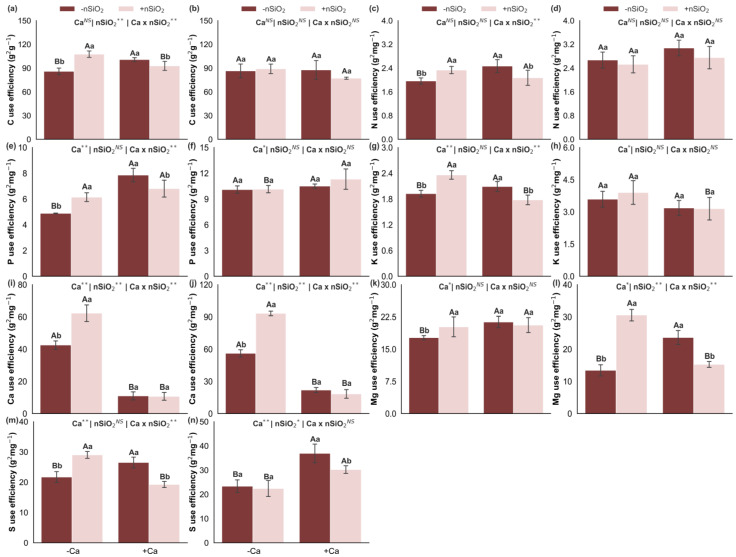
Carbon (**a**,**b**), nitrogen (**c**,**d**), phosphorus (**e**,**f**), potassium (**g**,**h**), calcium (**i**,**j**), magnesium (**k**,**l**), and sulfur (**m**,**n**) use efficiency in the shoot and roots of sugarcane plants grown under calcium deficiency (−Ca) or sufficiency (+Ca), combined with the absence (−nSiO_2_) or presence (+nSiO_2_) of silicon nanoparticle application. Bars represent means (*n* = 4) and error bars indicate the standard deviation (SD). Identical uppercase letters indicate no significant differences between Ca supply conditions, whereas identical lowercase letters indicate no significant differences between nSiO_2_ application conditions. *, ** and NS represent significance at 0.05, 0.01, and non-significant, respectively.

Under Ca deficiency, in the absence of nSiO_2_, reduced use efficiencies of C, N, P, K, and Mg were observed in the shoots, as well as of Mg and S in the roots, in addition to a reduction in S use efficiency in the shoots. In contrast, Ca use efficiency increased in both shoots and roots. The use efficiencies of C, N, P, and K in the roots were not affected (Figure 8). In Ca-deficient plants, the application of nSiO_2_, compared with its absence, increased the use efficiencies of C, N, P, K, Ca, Mg, and S in the shoots and of Ca and Mg in the roots, with no significant effects on the use efficiencies of C, N, P, K, and S in the roots (Figure 8). Under Ca-sufficient conditions, nSiO_2_ application resulted in decreased use efficiencies of C, N, P, K, and S in the shoots and of Mg in the roots, whereas the use efficiencies of Ca and Mg in the shoots and of C, N, P, K, and Ca in the roots did not show significant responses (Figure 8).

**Figure 9:**
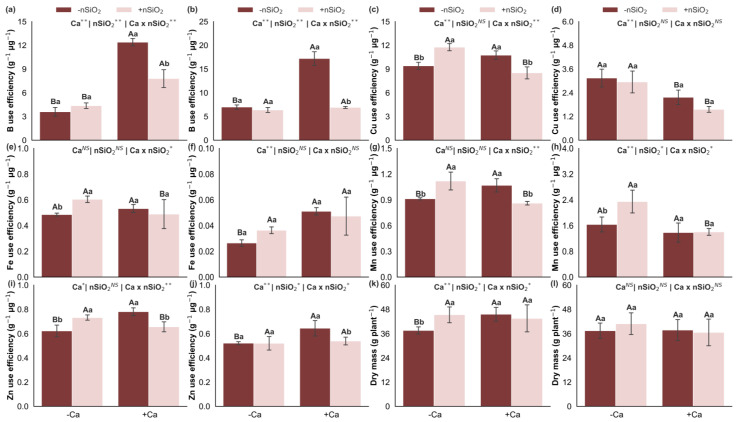
Boron (**a**,**b**), copper (**c**,**d**), iron (**e**,**f**), manganese (**g**,**h**) and zinc (**i**,**j**) use efficiency and dry mass (**k**,**l**) in the shoot and roots of sugarcane plants grown under calcium deficiency (−Ca) or sufficiency (+Ca), combined with the absence (−nSiO_2_) or presence (+nSiO_2_) of silicon nanoparticle application. Bars represent means (*n* = 4) and error bars indicate the standard deviation (SD). Identical uppercase letters indicate no significant differences between Ca supply conditions, whereas identical lowercase letters indicate no significant differences between nSiO_2_ application conditions. *, ** and NS represent significance at 0.05, 0.01, and non-significant, respectively.

Under Ca deficiency, in the absence of nSiO_2_, the use efficiencies of B, Cu, Mn, and Zn decreased in the shoots, and those of B, Fe, and Zn decreased in the roots, whereas Cu use efficiency increased in the roots. No significant response was observed for Fe use efficiency in the shoots or Mn use efficiency in the roots (Figure 9). Under Ca-deficient conditions, the application of nSiO_2_, relative to its absence, increased the use efficiencies of Cu, Fe, Mn, and Zn in the shoots and of Mn in the roots, with no effects on the use efficiencies of B in the shoots or of B, Cu, Fe, and Zn in the roots (Figure 9). Under Ca-sufficient conditions, nSiO_2_ application led to decreased use efficiencies of B, Cu, Mn, and Zn in the shoots and of B and Zn in the roots, whereas the use efficiencies of Fe in the shoots and of Cu, Fe, and Mn in the roots did not differ significantly (Figure 9).

### 2.7. Dry Mass

In the absence of nSiO_2_, shoot dry mass decreased under Ca deficiency; however, no significant response was observed for root dry mass (Figure 9k,l). The application of nSiO_2_, relative to its absence, increased shoot dry mass in Ca-deficient plants, whereas no significant effects were observed on shoot or root dry mass in Ca-sufficient plants or on root dry mass in Ca-deficient plants (Figure 9k,l).

### 2.8. Hierarchical Cluster Analysis (HCA)

**Figure 10:**
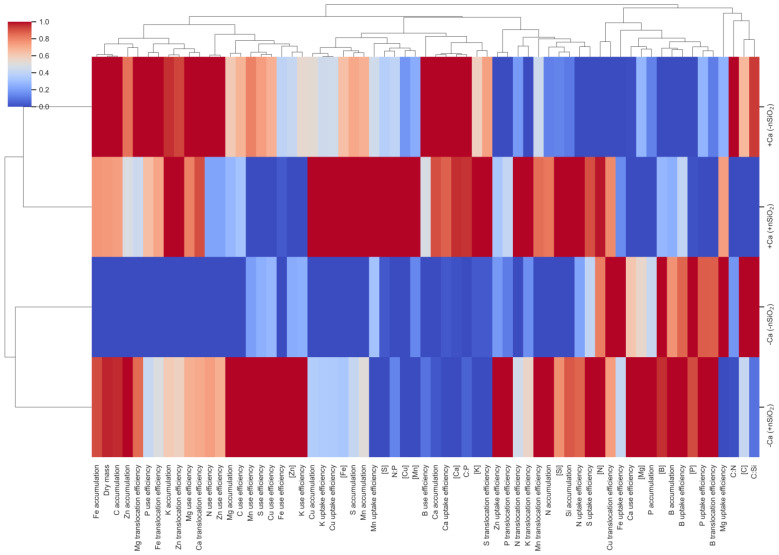
Hierarchical clustering analysis (HCA) displayed as heatmaps with dendrograms for variables measured in the shoots of sugarcane plants grown under calcium deficiency (−Ca) or calcium sufficiency (+Ca), combined with the absence (−nSiO_2_) or presence (+nSiO_2_) of silicon nanoparticle application. Variables include nutrient concentrations; nutrient accumulation (accum); uptake; translocation (transl); and use efficiency (use ef), dry mass and the stoichiometric ratios C:N, C:P, C:Si and N:P. Data were normalized (0–1); warmer colors indicate higher values, and cooler colors indicate lower values. Dendrograms depict similarity patterns among treatments and variables based on the multivariate response profiles.

Hierarchical cluster analysis (HCA) of shoot variables combined with nutrient uptake and translocation efficiencies revealed the formation of two major clusters primarily defined by Ca availability (+Ca vs. −Ca), whereas the addition of nSiO_2_ promoted an internal reordering within each Ca condition (Figure 10). The HCA indicated that a first cluster grouped Fe and Ca accumulation together with cation translocation variables (Mg translocation, Fe translocation, Zn translocation, and Ca translocation), suggesting coordination between shoot accumulation and the remobilization/redistribution of these elements (Figure 10).

A second cluster predominantly grouped nutrient use efficiencies (Zn use efficiency, C use efficiency, S use efficiency, Fe use efficiency, and K use efficiency) in association with K uptake efficiency (Figure 10). Subsequently, a module related to nutritional status and micronutrient homeostasis was observed, integrating Fe, S, and Cu concentrations, Mn accumulation, as well as B use efficiency, Ca uptake efficiency, and the C:P ratio (Figure 10). Finally, macronutrient translocation variables (S translocation, P translocation, and K translocation) clustered with indicators of elemental accumulation and balance (N accumulation, Si accumulation, S uptake efficiency, and Cu translocation) (Figure 10). This block was connected to a cluster including P accumulation, B accumulation, P concentration, and B translocation, together with the C:N and C:Si ratios; notably, C:Si showed an inverse pattern relative to Si accumulation (Figure 10).

**Figure 11:**
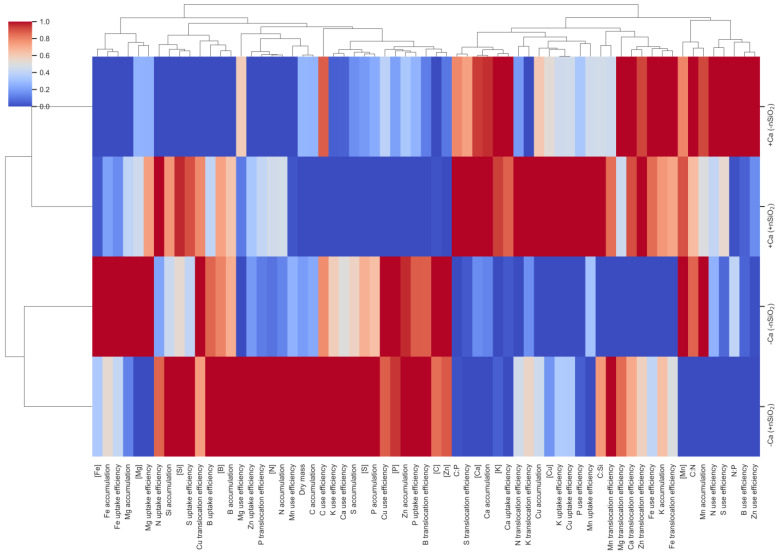
Hierarchical clustering analysis (HCA) displayed as heatmaps with dendrograms for variables measured in the roots of sugarcane plants grown under calcium deficiency (−Ca) or calcium sufficiency (+Ca), combined with the absence (−nSiO_2_) or presence (+nSiO_2_) of silicon nanoparticle application. Variables include nutrient concentrations, nutrient accumulation (accum), uptake, translocation (transl), and use efficiency (use ef), dry mass and the stoichiometric ratios C:N, C:P, C:Si and N:P. Data were normalized (0–1); warmer colors indicate higher values, and cooler colors indicate lower values. Dendrograms depict similarity patterns among treatments and variables based on the multivariate response profiles.

Hierarchical cluster analysis of root variables showed a clear separation of samples into two major clusters governed by Ca availability (Figure 11). Consistently, +Ca treatments clustered together and were distinct from −Ca treatments, indicating that Ca supply was the main factor structuring the multivariate response pattern in roots. Within each Ca condition, the presence of nSiO_2_ promoted internal reorganization of the clusters, suggesting a modulatory effect of Si on the nutritional profile and on nutrient acquisition and redistribution processes in the root system (Figure 11).

At the variable level, the dendrogram revealed well-defined functional modules. A first module grouped indicators related to Fe status and acquisition (Fe concentration and Fe uptake efficiency), which exhibited contrasting behavior between +Ca and −Ca and contributed strongly to group segregation (Figure 11). A second module aggregated variables associated with the Si signature and its integration with micronutrients, including Si and B concentrations and Cu translocation efficiency (Figure 11). In addition, a cluster concentrating growth/biomass production and use-efficiency variables was observed, such as N accumulation, dry mass, and C and Ca use efficiencies (Figure 11). In parallel, a rightmost module grouped attributes directly related to Ca and its associated transport, including Ca accumulation and Ca uptake and translocation efficiencies, connected to translocation and efficiency variables (K translocation, Fe use efficiency, Fe translocation) and stoichiometric ratios (C:N and C:Si) (Figure 11).

## 3. Discussion

### 3.1. Impact of Calcium Deficiency on the Modulation of C, N, and P Homeostasis and Nutritional Efficiency in Sugarcane Plants

The lower availability of Ca in the growth medium was directly reflected in reduced Ca concentrations in both shoots and roots, confirming that limited supply compromises the acquisition and accumulation of this nutrient in both compartments. This pattern is physiologically consistent, as Ca is predominantly absorbed as Ca^2+^, transported via the xylem, and exhibits limited mobility within plant tissues. Consequently, restrictions in Ca supply are rapidly translated into reduced tissue Ca concentrations, particularly in growing organs [19].

Calcium deficiency triggers structural and functional disorders because Ca is a key component of cell wall and membrane stability, acting through interactions with pectins and maintaining membrane selectivity and permeability [8]. Under low Ca conditions, the loss of this stabilizing function favors membrane instability and reduced integrity of young tissues and active roots, helping to explain why secondary nutritional disorders often emerge even when other nutrients are available [20].

In this context, the results indicate a reconfigured nutritional balance, showing that Ca deficiency led to higher P concentration in the shoots and increased concentrations of Si, C, Mg, and S in the roots, while the concentrations of several other nutrients remained stable (Figure 1 and Figure 2). This response reflects plant adjustments to maintain ionic and osmotic balance and sustain essential metabolic processes despite the structural limitations imposed by Ca deficiency. In addition, recent evidence demonstrates that Ca^2+^ signaling is closely linked to the regulation of nutrient uptake and transport, such that changes in Ca status can affect absorption and redistribution pathways of other elements [8].

Changes in C:N:P:Si stoichiometry further reinforce this framework, as Ca deficiency resulted in lower C:N and C:P ratios in the shoots and lower C:P and C:Si ratios in the roots (Figure 3), indicating elemental imbalance consistent with the idea that structural and growth limitations alter the dilution and concentration of elements within plant tissues. In line with recent studies in plant ecology and stoichiometry, Ca can act as a structuring factor of the multielemental profile, and when limiting, it reorganizes relationships among C, N, and P, with implications for allocation and efficiency [5,21].

These adjustments become more evident when nutrient accumulation and efficiencies are analyzed. Ca deficiency was associated with reduced accumulation of several nutrients in the shoots, including Si, C, K, Ca, and S, and with specific changes in the roots, such as reduced accumulation of K and Ca but increased accumulation of Si, Mg, and S (Figure 4). In parallel, Ca deficiency altered nutritional efficiencies, decreasing the uptake efficiencies of K, Ca, and Cu while increasing those of Mg, B, and Fe; reducing the translocation efficiencies of Ca, Mg, S, Fe, Mn, and Zn; and decreasing the use efficiencies of all macronutrients (except Ca) in the shoots and of Mg and S in the roots, while Ca use efficiency increased (Figure 8). Collectively, this pattern is consistent with a scenario in which Ca limitation compromises root and young tissue functionality and membrane integrity, and the plant attempts to compensate through reprogramming of uptake and transport processes, resulting in less efficient translocation and conversion of part of the nutrients into biomass [8,22].

These nutritional alterations converge into an integrated modulation that ultimately leads to reduced shoot dry mass, whereas root dry mass did not respond significantly (Figure 9k,l). This contrast suggests that Ca limitation imposed a stronger restriction on shoot growth, where the structural integrity of expanding tissues and membrane stability are particularly sensitive to low Ca availability. Overall, the results of this study reveal a logical sequence in which reduced Ca supply lowers tissue Ca concentrations, compromises structural integrity and homeostasis, triggers ionic and stoichiometric rebalancing, alters nutrient acquisition, transport, and use efficiencies, and culminates in shoot biomass loss.

### 3.2. Modulatory Role of Si Nanoparticles on C, N, and P Homeostasis and Nutritional Efficiency of Sugarcane Plants Under Ca Deficiency

The ability of silicon to modulate mineral nutrition and multielemental balance has been reported in several plant species. Studies in perennial crops, such as olive and grapevine, have shown that foliar applications of Si can alter plant nutritional composition, highlighting the role of this element as a modulator of mineral homeostasis across different agricultural systems [23,24]. Furthermore, similar effects on nutrient uptake, translocation, and use have also been reported with conventional silicon sources. In this context, the use of Si nanoparticles, as in the present study, may mainly increase the reactivity and delivery efficiency of the element to plants, thereby enhancing physiological mechanisms already associated with Si rather than necessarily introducing fundamentally new modes of action.

The application of nSiO_2_ increased Si concentration and accumulation in both shoots and roots of Ca-deficient plants (Figure 1 and Figure 4), demonstrating rapid incorporation of this element into plant tissues and its integration into the nutritional profile even under Ca-limited conditions. This increase in Si was accompanied by a reduction in the C:Si ratio in both shoots and roots, indicating a stoichiometric shift consistent with the concept that Si can act as a structural and functional component with lower metabolic cost compared to carbon-based compounds. Such a shift alters the balance between C and mineral elements and influences how plants organize their demands and allocations [25,26]. Recent evidence shows that Si can establish new stoichiometric relationships and modulate C:N:P balance across species, with direct implications for stress tolerance and resource-use efficiency [27,28,29].

From the perspective of C, N, and P homeostasis, the results indicate that under Ca deficiency, nSiO_2_ increased C and N accumulation in the shoots (Figure 4) and enhanced C, N, and P use efficiencies in the shoots (Figure 8), without consistently altering C:N, C:P, and N:P ratios (Figure 3). This pattern is particularly relevant, as it suggests a physiological adjustment associated more with improved efficiency—greater biomass return or metabolic productivity per unit of nutrient—than with simple growth dilution. Thus, plants maintained relatively stable C:N:P relationships while operating with higher efficiency, consistent with recent literature describing Si as a modulator of C assimilation, N metabolism, and P use organization under adverse conditions [29,30].

At the mechanistic level, three axes help explain this response: (i) carbon assimilation and allocation, (ii) nitrogen metabolism and utilization, and (iii) phosphorus acquisition and function [26,31]. Recent studies with nSiO_2_ report improvements in photosynthesis-related traits, maintenance of the photosynthetic apparatus, and metabolic performance, often associated with enhanced redox balance and preservation of cellular structures under stress. These effects tend to sustain higher C fixation and growth capacity even when a key nutrient such as Ca is limiting [32,33]. Future studies integrating metabolomic approaches may help further elucidate the biochemical pathways underlying silicon-mediated modulation of nutrient homeostasis and nutrient use efficiency.

Regarding N, nano-Si studies report associations between improved productivity or performance and parameters linked to N metabolism, including maintenance of assimilation pathways and functional N availability [27,33,34], consistent with the increased N use efficiency observed in shoots under Ca deficiency. For P, although tissue concentrations were not significantly altered by nSiO_2_ under Ca deficiency, improved P use efficiency in the shoots suggests more effective conversion of internal P into growth and biomass, possibly reflecting reorganization of the metabolic costs associated with P-dependent structures and processes. This is consistent with recent discussions of Si modulating nutrient use efficiency and C:N:P balance without necessarily increasing tissue concentrations [28,35].

Our results further showed that under Ca deficiency, nSiO_2_ increased the uptake efficiencies of N, K, S, Cu, and Zn and enhanced the translocation efficiencies of Ca, Mg, Fe, Mn, and Zn, while the uptake efficiencies of Ca, Mg, and Fe decreased. Such trade-offs are common in nutritional reprogramming and indicate that plants may prioritize pathways and elements more directly linked to immediate physiological performance, such as N for proteins and enzymes, K for osmotic regulation and enzyme activation, S for sulfur-containing and redox compounds, and key micronutrients [36,37], while restricting other fluxes due to physicochemical limitations, ionic competition, or transport adjustments. Recent literature on Si nanoparticles supports the idea that nano-Si can modulate nutrient acquisition and transport through changes in root architecture, exudation, and transporter activity, favoring reorganization of nutritional profiles under stress [38,39].

These modifications were directly reflected in biomass accumulation of Ca-deficient plants, as nSiO_2_ application increased shoot dry mass without significantly affecting root dry mass. This suggests a stronger functional gain in aboveground source–sink relationships—photosynthetic capacity, metabolic maintenance, and internal nutrient utilization—rather than direct stimulation of structural root biomass. Recent studies similarly associate nSiO_2_ applications with enhanced growth and productive performance, often linked to sustained physiological processes and improved resource-use efficiency under limiting conditions [33,40].

Overall, under Ca deficiency, nSiO_2_ acted as a modulator of nutritional functioning by intensifying Si integration into the system and shifting the C:Si ratio; sustaining C:N:P homeostasis without major disruptions in ratios while enhancing C, N, and P use efficiencies; reorganizing uptake and translocation patterns with selective gains in N, K, S, and strategic micronutrients; and translating these changes into increased shoot dry mass. Collectively, these results demonstrate that Si nanoparticles have the potential to reorganize elemental homeostasis and enhance nutritional efficiency in sugarcane plants under Ca deficiency, translating stoichiometric adjustments—particularly C:Si—nutrient accumulation, and uptake/translocation/use efficiencies into measurable gains in shoot biomass. This finding advances understanding of how Si-based inputs at the nanoscale can function as complementary tools to mitigate nutritional limitations in agricultural systems by promoting resource economy and improved nutrient-to-growth conversion.

### 3.3. Benefits of Si Nanoparticles in Optimizing C, N, and P Homeostasis and Nutritional Efficiency of Sugarcane Plants Under Ca Sufficiency

In Ca-sufficient plants, nSiO_2_ application acted primarily as a fine modulator of nutritional status and C:N:P balance rather than as a growth-promoting agent. This was evident because, even without significant biomass changes, nSiO_2_ increased Si and N concentrations in the shoots and Si concentration in the roots (Figure 1), indicating greater Si incorporation into the system and improved shoot N metabolism. This response is consistent with the capacity of Si—particularly in nanoparticulate form, with higher reactivity and gradual dissolution—to adjust physiological and biochemical processes related to N assimilation and use without necessarily translating these gains into dry mass increases when Ca is not limiting [41,42].

From a C:N:P homeostasis perspective, Ca sufficiency ensured structural and metabolic stability; within this context, the reduction in shoot C:N ratio observed with nSiO_2_ is consistent with greater N availability or assimilation per unit of C, favoring protein turnover, maintenance of chlorophyll and photosynthetic complexes, and enzyme activity related to primary metabolism [26]. Recent evidence shows that Si nanoparticles can stimulate photosynthesis, sugar metabolism, and hormonal pathways, thereby reconfiguring carbon partitioning and stoichiometric relationships, especially under non-limiting mineral conditions [41,43].

The most pronounced response under Ca sufficiency occurred in acquisition and redistribution efficiencies, as nSiO_2_ increased uptake efficiencies of N, K, Mg, S, B, Cu, and Mn (Figure 6) while also enhancing translocation efficiencies of K, S, and Mn (Figure 7). This pattern suggests that with adequate Ca—ensuring functional membranes and cell walls—nSiO_2_ can improve root system efficiency and xylem/phloem transport, possibly through adjustments in root architecture, transporter activity, and better coupling between uptake and canopy demand [29,44]. In sugarcane, Si effects on root function and nutrient acquisition have been discussed as mechanisms enhancing performance and physiological stability, even though many studies focus on stress conditions, reinforcing the role of Si as a modulator under non-limiting environments [25,45,46].

An important observation is that under Ca sufficiency, Ca uptake efficiency decreased with nSiO_2_ (Figure 6), and Mg and Fe translocation efficiencies tended to be lower (Figure 7). Mechanistically, this may reflect flux prioritization, whereby plants reduce Ca demand per unit biomass and redirect energy and transport capacity toward nutrients more directly linked to photosynthesis, osmotic balance, and metabolism (e.g., N, K, and S), while Si contributes to tissue stability and redox signaling at low intensity, adjusting physiological set points [26,43]. Recent studies on nSiO_2_ describe similar patterns of metabolic and carbohydrate regulation, where changes in energetic and antioxidant pathways occur without proportional growth responses when environmental conditions are already favorable [43,46,47].

Comparing the action of nSiO_2_ under Ca deficiency and sufficiency highlights distinct functional roles: under Ca deficiency, nSiO_2_ exhibited a compensatory profile associated with performance recovery and rebalancing of limiting processes; under Ca sufficiency, its effect was more homeostatic and optimizational, enhancing uptake and translocation efficiencies—particularly for N, K, S, and Mn—and adjusting stoichiometric relationships such as C:N, with limited translation into biomass gains due to the absence of stress. This distinction aligns with current models of Si action in grasses, in which Si acts as a stress attenuator under limiting conditions and as a regulator of efficiency and productivity under adequate conditions, especially in crops such as sugarcane [42,43,44,45,46,48].

Finally, our results indicate that under Ca sufficiency, nSiO_2_ can function as a fine-tuner of C:N:P homeostasis and nutritional efficiency even in the absence of primary Ca limitation, promoting consistent changes in nutrient acquisition and modifying stoichiometric relationships such as C:N and C:Si in the shoots.

## 4. Materials and Methods

### 4.1. Experimental Conditions

The experiment was conducted in a greenhouse at the Department of Soil Science, School of Agricultural and Veterinary Sciences, São Paulo State University (UNESP), Jaboticabal, SP, Brazil (21°15′19″ S, 48°19′21″ W; 615 m a.s.l.), during the spring season. Throughout the experimental period, the mean air temperature was 22.14 ± 10.0 °C and the mean relative humidity was 64.36 ± 7.0%.

Sugarcane seedlings were initially produced from stalks of the cultivar IAC05-5579 in polystyrene trays filled with vermiculite as substrate. After 30 days, the plants were transplanted into 4 L pots containing washed sand as substrate. The sand was thoroughly washed with deionized water until electrical conductivity reached approximately 10 μS cm^−1^., ensuring low mineral content. Subsequently, the material was treated with 0.1 mol L^−1^ HCl to remove potential contaminants and residual impurities, followed by rinsing with deionized water.

During the first 30 days after transplanting, plants received only deionized water. Thereafter, a nutrient solution based on [49] (Supplementary Table S1) was supplied, starting at 30% ionic strength, increased to 50% after five days and to 60% after an ad-ditional five days, remaining at this concentration for 30 days. To initiate nutrient omission treatments, the substrate was leached with deionized water until the electrical conductivity of the leachate was below 10 μS cm^−1^.

The experiment lasted approximately three months after transplanting the seedlings. This period was defined based on the progressive manifestation of calcium deficiency symptoms in treatments where the nutrient was omitted from the nutrient solution.

### 4.2. Treatments

The experiment was arranged in a completely randomized design in a 2 × 2 facto-rial scheme, with two Ca levels (deficient = 0.0 mmol L^−1^ and sufficient = 3.0 mmol L^−1^) and two Si conditions (absence = 0.0 mmol L^−1^; presence = 1.77 mmol L^−1^), with four replicates. At 60 days after transplanting, Ca was completely withdrawn from the nutrient solution in the Ca-deficient treatments.

Silicon was supplied in the form of SiO_2_ nanoparticles (specific surface area: 300 m^2^ g^−1^; particle size: 8.5–9.7 nm; Si concentration: 168.3 g L^−1^; pH 10.5). From 29 days after transplanting onward, nanoparticles were added daily to the nutrient solution. The solution pH was adjusted to 6.0 ± 0.5 using 0.1 mol L^−1^ NaOH or 0.1 mol L^−1^ HCl. Applications were carried out together with fertigation, using 250 mL of nutrient solu-tion per pot, adjusted to achieve a final Si concentration of 1.77 mmol L^−1^.

### 4.3. Analysis

#### 4.3.1. Biomass and Vegetative Growth

At the end of the experiment (89 days after transplanting), plant height, stem diameter, and number of leaves were measured using a graduated ruler, digital caliper, and manual counting, respectively. Biomass was separated into shoots and roots, followed by decontamination with a 0.1% detergent solution, 0.1% hydrochloric acid, and deionized water. Subsequently, samples were dried in a forced-air oven at 65 °C, and dry mass was determined using an analytical balance.

#### 4.3.2. Nutritional Analysis

Nutrient concentrations in shoot and root tissues were determined as follows. Carbon (C) content was quantified using the potassium dichromate (K_2_Cr_2_O_7_) oxida-tion method [50]. Total nitrogen (N) was determined after digestion in a heating block [51]. Phosphorus (P) was quantified after acid digestion in digestion tubes using a nitric–perchloric acid mixture, followed by colorimetric determination using a spectro-photometer [51]. Silicon (Si) determination was performed in 50-mL Falcon tubes using hydrogen peroxide (H_2_O_2_) and 50% sodium hydroxide (NaOH), followed by a col-orimetric reaction based on the formation of a yellow complex with ammonium mo-lybdate [(NH_4_)_6_Mo_7_O_24_] [52].Stoichiometric ratios (C:N, C:P, C:Si, and N:P) were calculated from nutrient concentrations in plant tissues. Nutrient accumulation was estimated as the product of dry mass and nutrient concentration. Uptake, translocation, and use efficiencies were calculated based on accumulation values according to Equations (1), (2), and (3), respectively [53,54].

(1) $$Nutrien\;uptake\;efficiency\;\left(g\;g^{-1}\right)=\frac{Total\;nutrient\;accumulation\;(g)}{Root\;dry\;mass\;(g)}$$

(2) $$Nutrient\;translocation\;efficiency\;\left(\%\right)=\frac{Shoot\;nutrient\;accumulation}{Total\;nutrient\;accumulation}\times 100$$

(3) $$Nutrient\;use\;efficiency\;\left(g^{2}\;g^{-1}\right)=\frac{{Dry\;mass}^{2}}{Nutrient\;accumulation}$$

### 4.4. Statistical Data Processing

Data normality was assessed using the Shapiro–Wilk test, whereas homogeneity of variances was evaluated with Levene’s test. Differences among treatments were analyzed by analysis of variance (ANOVA), adopting a significance level of *p* < 0.05. When significant effects were detected, means were compared using Tukey’s test, also considering *p* < 0.05. Principal component analysis (PCA) was applied to investigate the multivariate relationships among the evaluated variables. In addition, hierarchical clustering analysis was performed to classify treatments according to their similarity, using Euclidean distance as the measure of dissimilarity and the single-linkage method. All statistical analyses were conducted using the Python programming language, version 3.9.7 (Python Software Foundation).

## 5. Conclusions

Calcium deficiency directly impaired the nutritional status of sugarcane, resulting in lower Ca concentrations in both shoots and roots and triggering a disruption of mineral homeostasis, which was reflected in altered C:N:P stoichiometry; nutrient accumulation; and nutrient uptake, translocation, and use efficiencies. This set of changes ultimately led to reduced shoot dry mass, whereas root dry mass did not show a significant response, indicating a greater sensitivity of the shoot to Ca limitation.

The application of nSiO_2_ to Ca-deficient plants acted as a compensatory strategy, promoting greater integration of Si into the system, stoichiometric adjustments—particularly in the C:Si ratio—and coordinated improvements in nutritional performance, with increases in selected nutrient accumulations and efficiencies and, most importantly, enhanced shoot dry mass. In contrast, under Ca-sufficient conditions, nSiO_2_ application exerted a predominantly homeostatic and optimizational effect, adjusting nutrient uptake and redistribution and modulating C:N:P stoichiometric relationships without translation into biomass gain, indicating that its benefits are more evident under conditions of nutritional stress.

## Data Availability

Data will be made available on request.

## Supplementary Materials

The following supporting information can be downloaded at: https://www.mdpi.com/article/10.3390/plants15060971/s1, Table S1: Nutrient solution composition proposed by Hoagland and Arnon (1950).

## Abbreviations

The following abbreviations are used in this manuscript:

nSiO_2_	Silicon dioxide nanoparticles
μS cm^−1^	Microsiemens per centimeter
C:N	Carbon-to-nitron ratio
C:P	Carbon-to-phosphorus ratio
C:Si	Carbon-to-silicon ratio
N:P	Nitrogen-to-phosphorus ratio
HCA	Hierarchical Cluster Analysis
SD	Standard deviation
HCl	Hydrochloric acid
NaOH	Sodium hydroxide
$H_{2}O_{2}$	Hydrogen peroxide
$K_{2}{{Cr}_{2}O}_{7}$	Potassium dichromate
(NH_4_)_6_Mo_7_O_24_	Ammonium molybdate
UNESP	São Paulo State University
APC	Article Processing Charge
DOI	Digital Object Identifier
