# Supplementary material for: Silicon Nanoparticles Modulate C:N:P Homeostasis and the Efficiencies of Nutrient Uptake, Translocation, and Use in Sugarcane Under Calcium Deficiency and Sufficiency
Source: Plants (Basel). 2026 Mar 21;15(6):971. doi: 10.3390/plants15060971 (PMC13030359; doi:10.3390/plants15060971)
Supplement: Supplementary file 1 [file plants-15-00971-s001.zip › plants-4175645-supplementary.pdf]

Table S1. Composition of the nutrient solution proposed by Hoagland and Arnon (1950).

**Reagents**	-Ca		+Ca	
	-Si	+Si	-Si	+Si
	mM			
KH_2PO_4	0.600		0.600	
KNO_3	3.000		3.000	
$Ca\ (NO_3)_2.4H_2O$	-		3.000	
$MgSO_4.7H_2O$	1.200		1.200	
H_3BO_3	0.0276		0.0276	
$MnCl_2.2H_2O$	0.0054		0.0054	
$ZnSO_4.7H_2O$	0.00045		0.00045	
CuCl2	0.00018		0.00018	
$H_2MoO_4H_2O$	0.000066		0.000066	
Fe-EDTA	0.11206		0.11206	
